# Highly complex magnetic behavior resulting from hierarchical phase separation in AlCo(Cr)FeNi high-entropy alloys

**DOI:** 10.1016/j.isci.2022.104047

**Published:** 2022-03-11

**Authors:** Qianqian Lan, András Kovács, Jan Caron, Hongchu Du, Dongsheng Song, Sriswaroop Dasari, Bharat Gwalani, Varun Chaudhary, Raju V. Ramanujan, Rajarshi Banerjee, Rafal E. Dunin-Borkowski

**Affiliations:** 1 Ernst Ruska-Centre for Microscopy and Spectroscopy with Electrons and Peter Grünberg Institute, Forschungszentrum Jülich, 52425 Jülich, Germany; 2 School of Materials Science and Engineering, Tsinghua University, Beijing 100086, China; 3 Central Facility for Electron Microscopy, RWTH Aachen University, 52074 Aachen, Germany; 4 Department of Materials Science and Engineering, University of North Texas, Denton, TX 76201, USA; 5 School of Materials Science and Engineering, Nanyang Technological University, Singapore 639798, Singapore

**Keywords:** Condensed matter physics, Phase transitions, Magnetism

## Abstract

Magnetic high-entropy alloys (HEAs) are a new category of high-performance magnetic materials, with multicomponent concentrated compositions and complex multi-phase structures. Although there have been numerous reports of their interesting magnetic properties, there is very limited understanding about the interplay between their hierarchical multi-phase structures and the resulting magnetic behavior. We reveal for the first time the influence of a hierarchically decomposed B2 + A2 structure in an AlCo_0.5_Cr_0.5_FeNi HEA on the formation of magnetic vortex states within individual A2 (disordered BCC) precipitates, which are distributed in an ordered B2 matrix that is weakly ferromagnetic. Non-magnetic or weakly ferromagnetic B2 precipitates in large magnetic domains of the A2 phase, and strongly magnetic Fe-Co-rich interphase A2 regions, are also observed. These results provide important insight into the origin of coercivity in this HEA, which can be attributed to a complex magnetization process that includes the successive reversal of magnetic vortices.

## Highlights

• Al(Co,Cr)FeNi alloys consist of hierarchically decomposed B2 + magnetic A2 phases
• In AlCoFeNi, nanosized phases form magnetic domains with small angle alignment
• In AlCo_0.5_Cr_0.5_FeNi, B2 region contains A2 magnetic vortices and A2 with B2 inclusions
• The switching behavior of the magnetic microstructure is related to soft magnetism

## Graphical abstract

**Figure:**
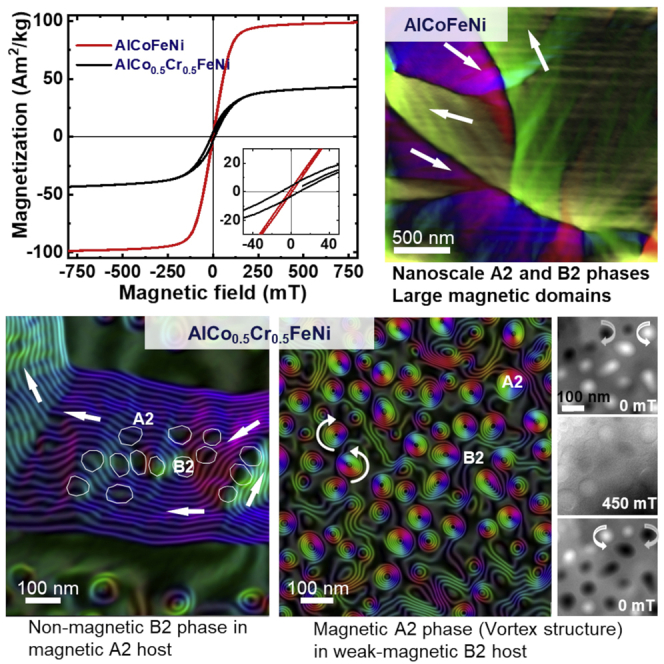

## Introduction

Magnetic materials are essential in a wide and growing variety of industrial, commercial, and residential applications, including rotating electrical machines, electric vehicles, wind turbines, transformers, power converters, electronic article surveillance systems, magnetically coupled devices, sensors, and high-frequency electromagnetic devices (Chaudhary et al., 2020; McHenry et al., 1999)^,^. To illustrate the importance of magnetic materials, we note that the energy utilization of electrical machines is a significant fraction of the total energy consumption in the world (Weiss et al., 2020). Even a 1% increase in their efficiency would have a great impact on energy efficiency and on the reduction of CO_2_ emissions. Hence, there is considerable interest in the development of improved magnetic materials, such as magnetic high-entropy alloys (HEAs), for next-generation magnets.

HEAs contain combinations of multiple principal elements in high concentration (Miracle and Senkov, 2017; Yeh et al., 2004). They are attracting significant attention because their rich compositional variety and phase space provide opportunities for discovering alloys that have outstanding mechanical and functional properties (George et al., 2019; Senkov et al., 2015). HEAs can form single phases, comprising random solid solutions of their constituent elements. However, local variations in chemical composition and short-range order have a strong effect on dislocation movement and planar defect energy, leading to increased yield strength without compromising strain hardening and tensile ductility (Ding et al., 2019; Xu et al., 2018), just as for numerous multi-phase alloys and composites in which increased heterogeneity results in improvements in properties (Ma and Wu, 2019; Ma et al., 2018). Considering the huge number of possible combinations of elements and processing conditions that can lead to the formation of secondary phases, the possibilities for developing multi-phase systems with different functionalities are enormous (Yan and Zhang, 2020).

Although the mechanical properties of many HEAs are well studied and can be superior to those of conventional alloys, much less work has been performed to obtain an in-depth understanding of their magnetic properties. Ferromagnetic HEAs have good mechanical properties and a wide range of tunable magnetic properties, ranging from soft to semihard to hard (Chaudhary et al., 2018; Dasari et al., 2020; Na et al., 2021). The formation of compositional heterogeneities and secondary phases can also alter the magnetic properties of HEAs. In general, ferromagnetic HEAs that have face-centered cubic (FCC) and body-centered cubic (BCC) structures are based on Fe, Co, and Ni. An exception is the hexagonal-close-packed (HCP) HoDyYGdTb HEA, which has a rich and complex magnetic phase diagram below room temperature (Lužnik et al., 2015). The magnetic characteristics of HEAs are highly sensitive to the compositions and morphologies of the constituent phases over various length scales (Cieslak et al., 2020; Jung et al., 2019; Miracle and Senkov, 2017; Vrtnik et al., 2018). For example, the addition of paramagnetic or antiferromagnetic elements can induce phase segregation and decrease saturation magnetization *M*_S_ (Jung et al., 2019), the addition of 25% Cr to FeCoNi can make the resulting FeCoNiCr alloy paramagnetic (Lucas et al., 2011), and the introduction of Mn to FeCoNiCr can eliminate the energy difference between FCC and HCP structures, resulting in magnetic frustration (Niu et al., 2018).

An in-depth understanding of structure-magnetism correlations in HEAs has been limited by a lack of appropriate methods to characterize their phases and properties at the nanoscale and beyond. Most magnetic property studies of HEAs are based on conventional magnetization hysteresis loop (*M*-*H*) measurements of bulk forms of HEAs, and are unable to reveal local magnetic states and interactions between the constituent phases. The magnetic properties of highly heterogeneous alloys, in which each phase displays chemical and topological disorder, cannot be described as a composition-weighted average of the magnetic properties of the constituent phases (Cieslak et al., 2020; Vrtnik et al., 2018). Specifically, the origin of the dramatic difference between the magnetic properties of AlCoFeNi and AlCo_0.5_Cr_0.5_FeNi HEAs is unexplored. An experimental understanding of the relationship between the local structure and magnetic texture of AlCoFeNi and AlCo_0.5_Cr_0.5_FeNi HEAs is crucial to control their properties.

Here, we use three-dimensional (3D) atom probe tomography (APT) and transmission electron microscopy (TEM) methods, including the Fresnel mode of Lorentz TEM and off-axis electron holography (EH), to investigate the influence of complex hierarchical phase separation of the A2 phase (disordered BCC) (Badjuk et al., 1974; Buschow et al., 1983; Ellis, 1941) and the CsCl-structured B2 phase (ordered BCC) (Dutchak and Chekh, 1981) on local variations in the magnetic behavior of AlCo(Cr)FeNi HEAs. We study the local magnetic characteristics of the individual phases, and provide quantitative measurements of saturation magnetic induction. Our results provide local experimental measurements of correlations between structure and magnetic properties in HEAs with nm spatial resolution. This information is essential for understanding complex magnetism in multi-phase AlCo(Cr)FeNi alloys, as well as for the design of new HEAs with unique tailored magnetic properties.

## Results

We first report the magnetic properties of AlCoFeNi and AlCo_0.5_Cr_0.5_FeNi bulk HEAs, followed by TEM studies of the structure and magnetism of AlCoFeNi. The multi-length-scale structure of AlCo_0.5_Cr_0.5_FeNi is then investigated. Two regions (R1, R2) are identified, within which a heterogeneous phase distribution is observed. The static and dynamic magnetic behavior of R1 and R2, as well as the phases present in these regions, is studied in detail.

### Magnetic properties of heat-treated AlCoFeNi and AlCo_0.5_Cr_0.5_FeNi bulk HEAs

**Figure 1:**
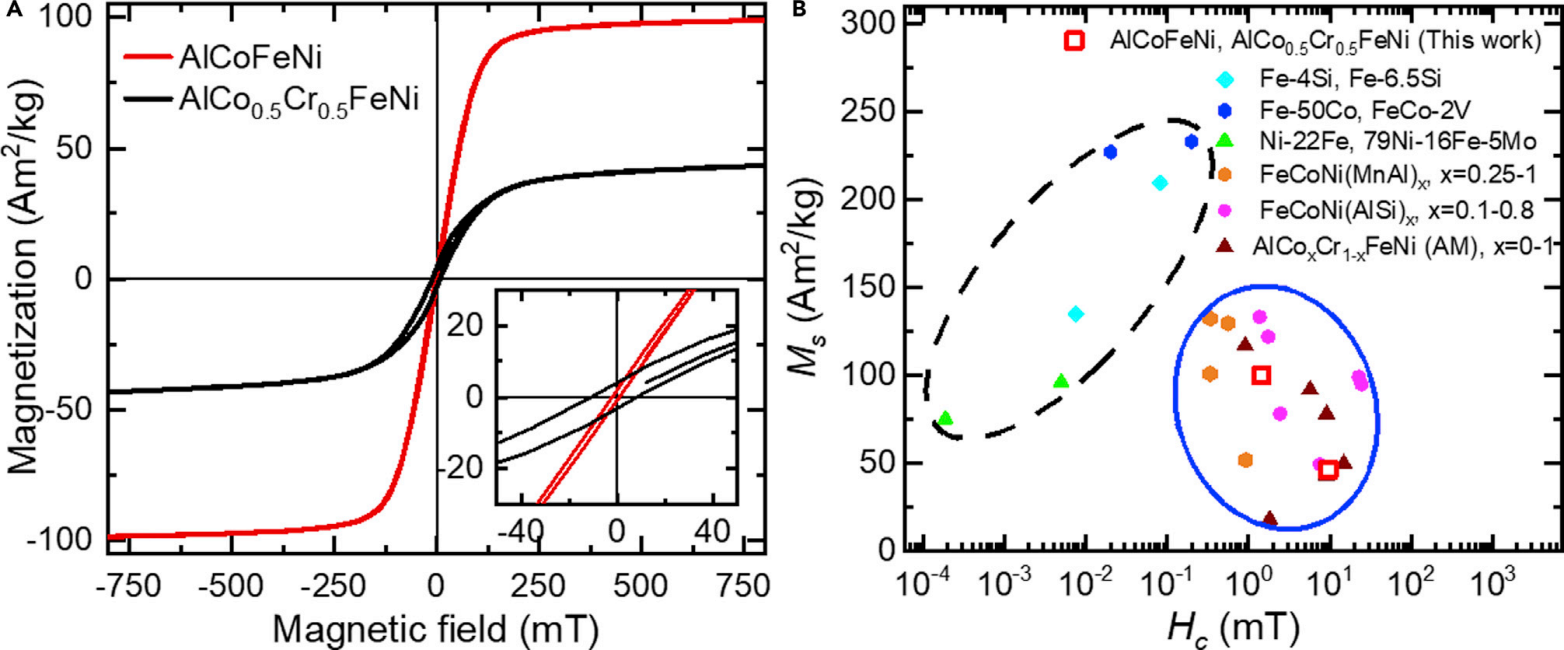
Magnetic properties of AlCoFeNi and AlCo_0.5_Cr_0.5_FeNi HEAs (A) Magnetization (*M*) vs applied magnetic field (H) measured at a temperature of 300 K for AlCoFeNi and AlCo_0.5_Cr_0.5_FeNi HEAs. The inset shows a magnified view of the central part of the hysteresis loop. (B) Saturation magnetization *M*_S_ and coercivity *H*_C_ of selected soft magnetic materials (dashed black oval) and HEAs (solid blue oval) (Dasari et al., 2020). The AlCoFeNi and AlCo_0.5_Cr_0.5_FeNi HEAs studied here are represented by red squares.

Figure 1A shows magnetization vs magnetic field (*M*-*H*) hysteresis loops measured at 300 K for heat-treated AlCoFeNi and AlCo_0.5_Cr_0.5_FeNi. The loops suggest soft ferromagnetic properties. AlCoFeNi has a saturation magnetization (*M*_*S*_) of 99.8 Am^2^/kg and a coercivity (*H*_*c*_) of 1.45 mT. When half of the Co is replaced by Cr in the AlCo_0.5_Cr_0.5_FeNi HEA, *M*_*S*_ decreases to 46.2 Am^2^/kg, while *H*_*c*_ increases to 9.56 mT. The HEAs are almost fully magnetically saturated at fields of 500 mT. Figure 1B shows a comparison of *M*_S_ and *H*_C_ for AlCoFeNi and AlCo_0.5_Cr_0.5_FeNi HEAs with other HEAs and benchmark soft magnetic materials (Borkar et al., 2017; Chaudhary et al., 2021; Dasari et al., 2020; Li et al., 2017; Zhang et al., 2013). The magnetic properties of the HEAs that we have studied are similar to others. The HEAs have higher values of *H*_C_ than those of some soft magnetic materials (e.g., Permalloy, Hiperco). We do not claim that their soft magnetic properties are superior. Instead, we focus on the reasons for the substantial difference in magnetic properties when 50% Co is replaced by Cr in AlCo_0.5_Cr_0.5_FeNi. For example, permeability is low (9.4 for AlCoFeNi and 3.9 for AlCo_0.5_Cr_0.5_FeNi), which is also true for other HEAs (Gao et al., 2018). As mentioned earlier, the application space for such HEA requires reasonable magnetic properties and mechanical properties which are not available in conventional magnetic materials.

### Microstructural and magnetic properties of heat-treated AlCoFeNi

**Figure 2:**
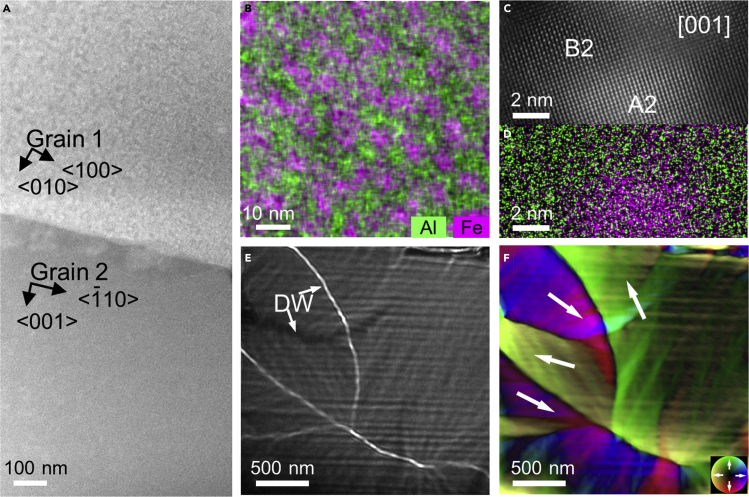
Structural and magnetic texture of the AlCoFeNi HEA (A) HAADF STEM image of two grains, with grain 1 aligned to a zone axis. The grain orientations are indicated. (B) Combined Al + Fe elemental map obtained from grain 1 using STEM EDXS imaging. (C and D) Atomic-resolution HAADF STEM image and (D) corresponding EDX map. (E) Overfocus (100 *μ*m) Fresnel defocus image recorded from grain 2. (F) Corresponding magnetic induction map reconstructed using the transport-of-intensity equation. The color wheel indicates the direction and magnitude of the projected in-plane magnetic induction.

The AlCoFeNi HEA has a polycrystalline structure with a grain size of above 100 *μm*. Figure 2A shows an HAADF STEM image of a grain boundary region between two adjacent AlCoFeNi grains. Grain 1 was aligned in the electron microscope to the closest crystallographic zone axis, at which an (001) direction was parallel to the electron beam direction. In this projection, chemically sensitive contrast reveals inhomogeneities. 3D APT studies of AlCoFeNi have previously revealed the presence of nm-sized Fe-Co-rich A2 precipitates in an Al-Ni-rich B2 matrix (Chaudhary et al., 2018). Figure 2B shows Al and Fe elemental distributions measured using energy-dispersive X-ray spectroscopy (EDXS) in grain 1, confirming the presence of compositional variations. The average sizes of the Al-rich and Fe-rich regions are approximately 10 nm. Figures 2C and 2D show an atomic-resolution image and a corresponding elemental map of the Al-rich B2 phase and the Fe-rich A2 phase. The structures are mostly coherent, with occasional dislocations at the interface due to the lattice misfit.

The magnetic domain structure at remanence was initially studied using Fresnel defocus imaging in Lorentz mode in magnetic-field-free conditions. Figure 2E shows an overfocus Fresnel image recorded from grain 2. This region contains large magnetic domains, which are separated by magnetic domain walls that appear as black or white lines. The ripple-like contrast in the domains originates from small-angle magnetization variations. Figure 2F shows a representation of the projected in-plane magnetic induction obtained from two defocused images using the transport-of-intensity equation (Gureyev and Nugent, 1997; Gureyev et al., 1995). The magnetic domain configuration was observed to rearrange upon applying a magnetic field as small as 5 mT perpendicular to the specimen using the microscope objective lens. This observation suggests isotropic soft ferromagnetic behavior, in which the phase-separated structure is not strong enough to pin domain walls.

### Multi-length-scale structure of AlCo_0.5_Cr_0.5_FeNi

**Figure 3:**
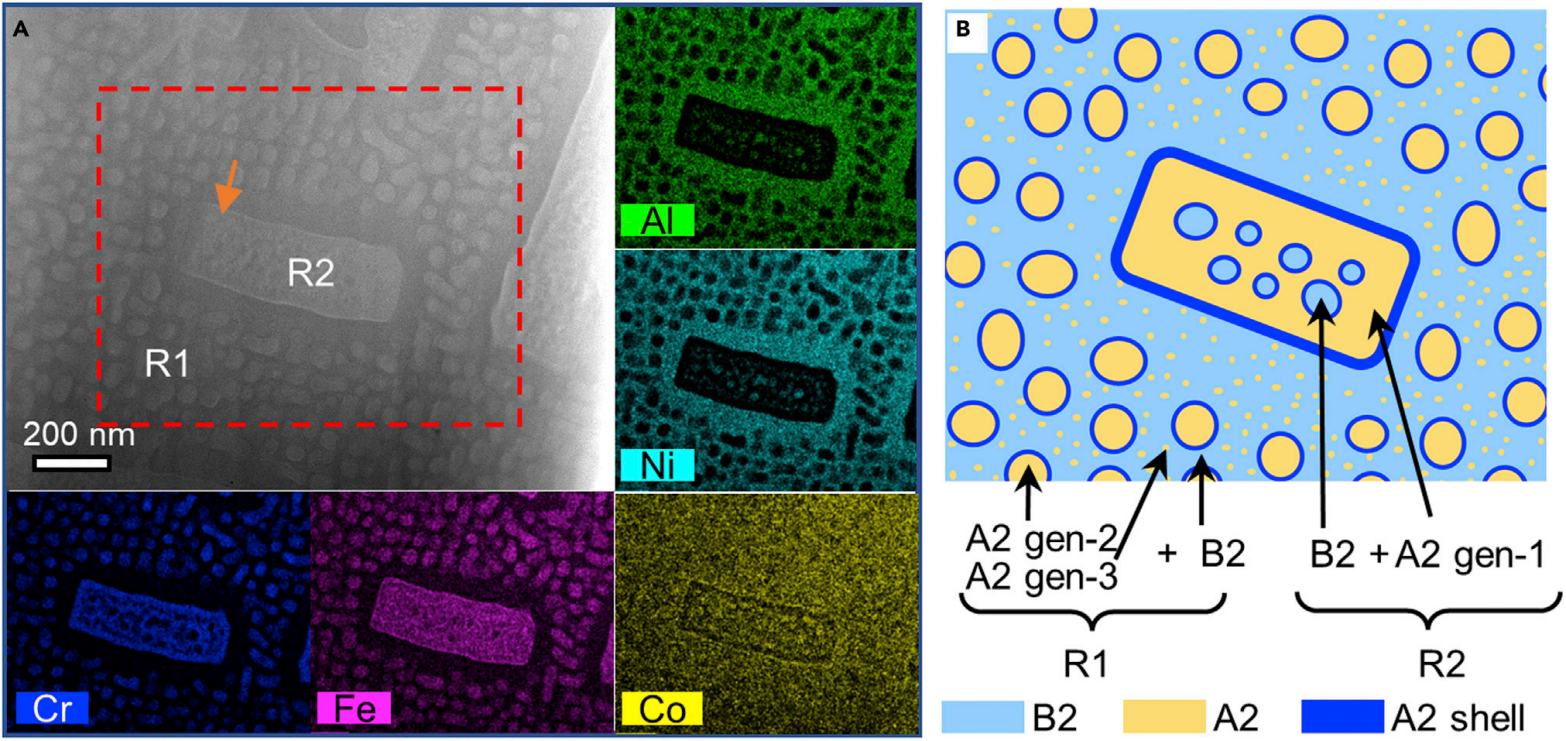
Phase separation in the AlCo(Cr)FeNi HEA (A) HAADF STEM image of the multi-scale hierarchical B2 and A2 structure, containing characteristic R1 and R2 regions. EDXS elemental maps of Al, Cr, Fe, Co and Ni recorded from the marked rectangular area are shown around the main frame. An orange arrow marks an A2 shell around an R2 island. (B) Schematic diagram of the R1 and R2 phase arrangements. In the R1 phase, fine A2 gen-3 and medium A2 gen-2 precipitates form in a B2 matrix. The A2 gen-2 precipitates are covered by an A2 shell. In region R2, B2 precipitates form in an A2 gen-1 matrix covered by an A2 shell.

Figure 3A shows that the annealed AlCo_0.5_Cr_0.5_FeNi HEA specimen has a strikingly different structure from that of the AlCoFeNi alloy, comprising an Al-Ni-Co-rich B2 phase, Fe-Cr-Co-rich A2 regions, and their combinations. The different length scales of the A2 phases are referred to here as a) coarse A2 gen-1 (with an average size of 1–5 *μ*m), b) medium-scale A2 gen-2 (50-150 nm), and c) fine-scale A2 gen-3 (<10 nm). Two characteristic regions are labeled R1 (A2 gen-2 + A2 gen-3 + B2 matrix) and R2 (A2 gen-1 + B2 matrix). An additional A2 phase (A2 shell) is observed between the B2 and A2 phases (see below), taking the form of a few-nm-thick shell around the A2 gen-1 and A2 gen-2 phases. Figure 3B shows a schematic diagram of the constituent phases and regions in the AlCo_0.5_Cr_0.5_FeNi HEA specimen.

**Figure 4:**
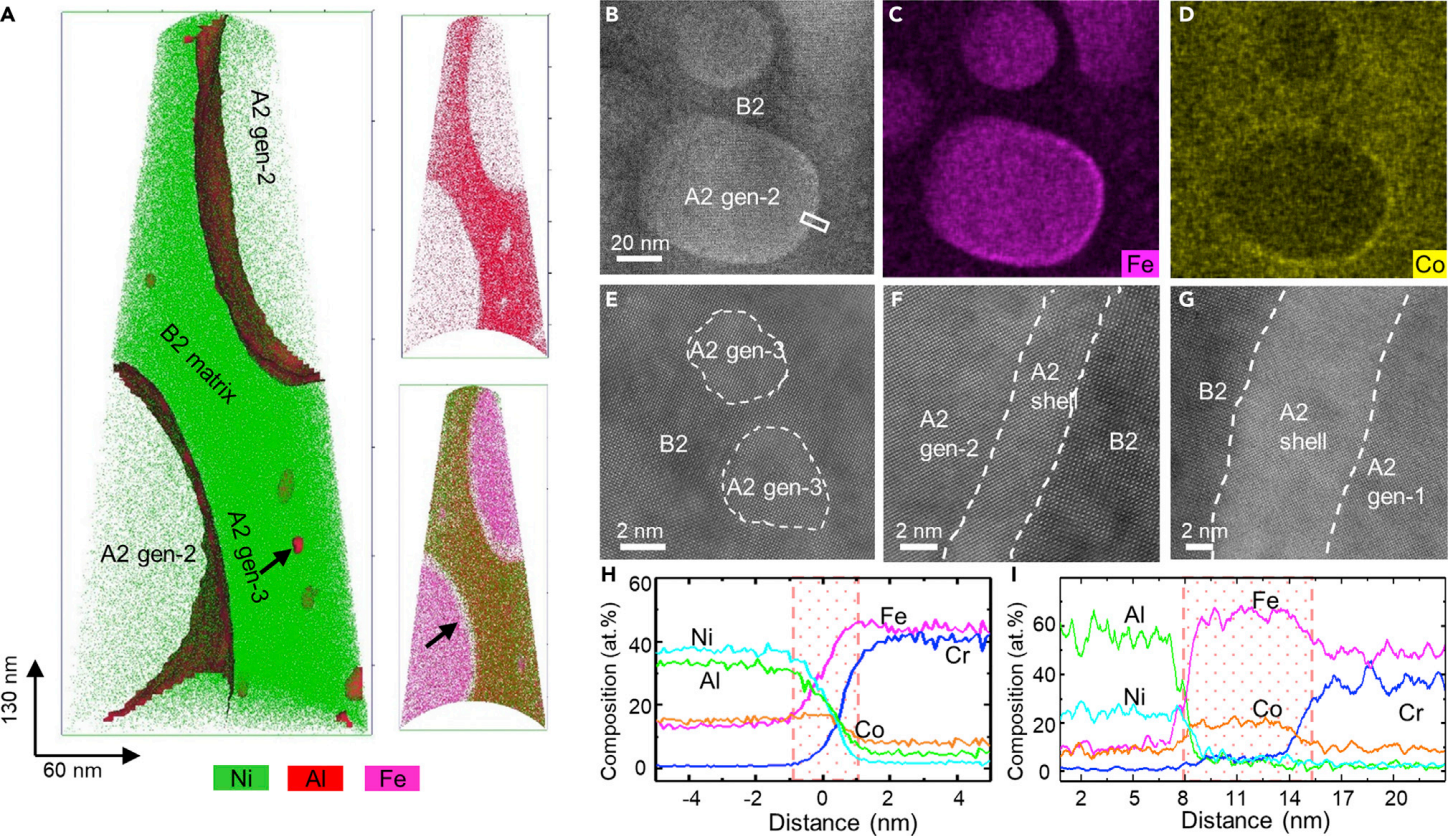
Microstructure and chemical composition of the annealed AlCo_0.5_Cr_0.5_FeNi HEA (A) 3D APT reconstruction of A2 gen-2 and gen-3 precipitates in a B2 matrix in region R1. An Fe-Co-rich shell forms around the A2 gen-2 precipitates. The colors represent Al (red), Ni (green), and Fe (magenta). (B–D) HAADF STEM image and corresponding Fe and Co elemental maps measured using EDXS imaging. Fe and Co enrichment is evident in the interface region of the two phases. (E–G) Atomic-resolution HAADF STEM images of A2 gen-3 in the B2 matrix, the A2 shell around the A2 gen-2 precipitates (marked by a white rectangle in B), and the A2 shell around an A2 gen-1 island (marked by an orange arrow in Figure 3A), respectively. (H and I) Compositional profiles across the A2 shells around the A2 gen-2 phase and the A2 gen-1 phase, respectively. Fe and Co enrichment is observed in the shells.

Figure 4 shows TEM and APT measurements of the shell A2 phase. Figure 4A shows an APT reconstruction of the elemental distribution in a needle-shaped specimen of region R1 in the AlCo_0.5_Cr_0.5_FeNi HEA, revealing small Fe-Cr-rich A2 gen-3 precipitates and large A2 gen-2 precipitates surrounded by Fe-Co-rich A2 shells in a B2 matrix. Figure S1 shows the chemical composition of the shell around the A2 gen-2 precipitates in details.

Figures 4B–4D show an HAADF STEM image and corresponding Co and Fe EDXS elemental maps of the shell region. The enhancement in Co and Fe at the edges of the A2 gen-2 precipitate appears to be discontinuous, perhaps because of the geometry of the thin TEM specimen, from which part of the precipitate may have been removed by ion milling. Figure 4E shows an atomic-resolution HAADF STEM image of A2 gen-3 precipitates in the B2 matrix. Figures 4F and 4G show atomic-resolution HAADF STEM images of the A2 structure of the shell, whose thickness is 2 nm around the A2 gen-2 precipitates and 7.5 nm around the A2 gen-1 island. The measurements are consistent between the APT result and the EDXS images shown in Figures 4H and 4I. The A2 shell is rich in Fe and Co, whereas little or no Al or Cr is present inside it. Atomic-resolution HAADF STEM images of the interface region are presented in Figure S3.

### Magnetic properties of AlCo_0.5_Cr_0.5_FeNi (I) – A2 gen-2 precipitates in the B2 matrix (region R1)

#### Static magnetic behavior

Highly heterogeneous systems, in which each region contains both chemical and topological disorder, such as heat-treated AlCo_0.5_Cr_0.5_FeNi HEAs, typically have complex magnetic properties in the constituent phases that cannot be understood from bulk magnetic measurements alone. We studied the local magnetic properties of the A2 and B2 phases in the R1 and R2 regions of the AlCo_0.5_Cr_0.5_FeNi specimen using Fresnel defocus imaging and off-axis EH. The latter technique provides a direct quantitative measurement of the phase shift of the electron wave that interacted with the specimen, from which the local magnetic state of the region of interest can be determined with nm spatial resolution (Dunin-Borkowski et al., 2019). The total electron-optical phase shift contains electrostatic and magnetic contributions that need to be separated to measure the magnetic field distribution in the specimen. In the absence of electron-beam-induced charging, the electrostatic contribution to the phase shift is proportional to the projected mean inner potential (MIP) of the specimen, which depends on its composition, density, and ionicity.

**Figure 5:**
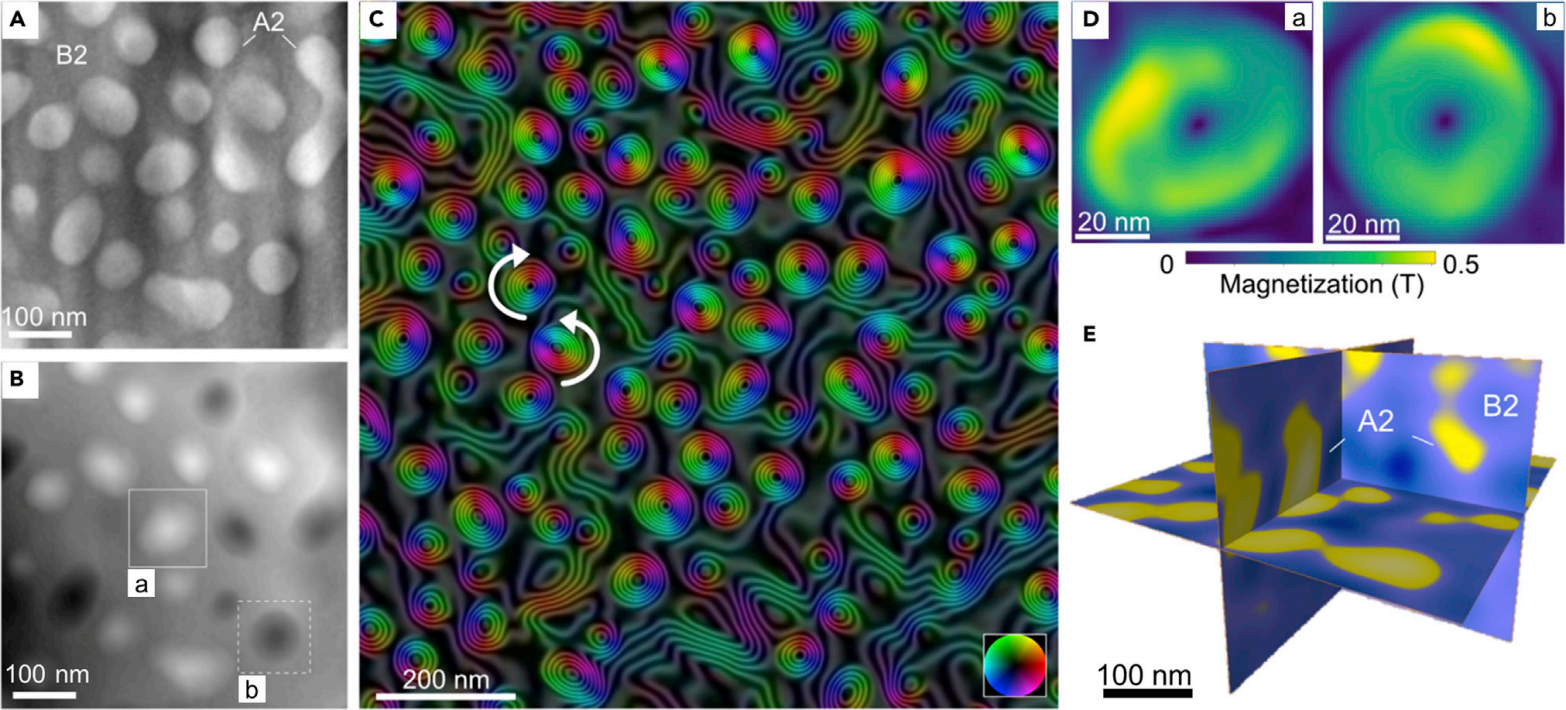
Static magnetic behavior of region R1 containing A2 precipitates in a B2 matrix (A and B) Mean inner potential and (B) magnetic contributions to the phase shift recorded using off-axis EH with the specimen in a magnetic remanent state. The magnetic phase shift (*φ*_*M*_) in the A2 gen-2 precipitates is either bright or dark, as a result of the presence of a magnetic vortex in each precipitate. The marked A2 gen-2 precipitates (A and B) are further analyzed in (D). (C) Large-field-of-view magnetic induction map derived from the magnetic contribution to the phase shift, showing clockwise and counterclockwise magnetic vortex states in the A2 precipitates. The contour spacing is 2*π*/24 rad. (D) Projected in-plane magnetization (*M*_*xy*_) in the A2 precipitates marked in (B) determined from the magnetic contribution to the phase shift using model-based iterative reconstruction. An upper limit for the diameter of the magnetic vortex core, which has an out-of-plane magnetic field orientation, is 8 nm. (E) Sections showing embedding of A2 precipitates in the B2 matrix extracted from a tomographic reconstruction obtained from a tilt series of ADF STEM images.

Figure 5A shows the MIP contribution to the electron-optical phase shift in region R1 measured using off-axis EH at magnetic remanence. In this image, the A2 gen-2 precipitates, which have close-to-spherical morphologies and diameters of between 50 and 120 nm, appear brighter than the surrounding B2 matrix, as they have a higher mean atomic number per unit volume. Figure 5B shows the corresponding magnetic contribution to the phase shift *φ*_*M*_, which provides a measure of the in-plane component of the magnetic induction within and outside the specimen integrated in the electron beam direction (Kovács and Dunin-Borkowski, 2018). Bright or dark contrast is visible within the boundaries of the A2 gen-2 precipitates, which are each surrounded by a thin Fe-Co-rich A2 shell. Figure 5C shows a magnetic induction map obtained by generating contour lines and colors from the magnetic contribution to the phase shift and its gradient, respectively. This image reveals that each A2 precipitate contains a magnetic vortex, with the magnetic field rotating either clockwise or counterclockwise. Similar 3D magnetic vortex states have been observed in sub-100-nm spherical Fe-Ni particles without strong magnetocrystalline anisotropy (Hÿtch et al., 2003; Kim et al., 2014). In region R1, the A2 precipitates are well separated by the B2 matrix, preventing dipolar interactions between individual crystals. The ratio of clockwise to counterclockwise magnetic vortices is approximately 1:1 at remanence after saturating the specimen using the microscope objective lens, suggesting the formation of energetically independent magnetic states that are only weakly coupled to the surrounding phases. The magnetic vortices are not associated with significant stray magnetic fields that could be measured at remanence using either bulk magnetometry or surface-sensitive magnetic characterization techniques.

A model-based iterative reconstruction algorithm (Caron, 2017) was used to convert the measured magnetic phase images *φ*_*M*_ into maps of projected in-plane magnetization *M*_*xy*_, as shown in Figure 5D for the two A2 gen-2 precipitates marked in Figure 5B. The magnetization direction of the vortex core is parallel to the incident electron beam direction in the center of each precipitate and does not contribute to the projected in-plane magnetization map in this region. In general, each magnetic vortex core can point either up or down magnetically, irrespective of the vortex rotation direction (Shinjo et al., 2000). The magnetization direction of the vortex core cannot be detected from these images, as EH is sensitive to the component of the magnetic field that is perpendicular to the incident electron beam direction. An upper limit for the magnetic vortex core diameter was measured to be ∼8 nm by fitting a Gaussian function to the projected in-plane magnetization distribution. On the assumption that the specimen is magnetically active through its entire thickness, the magnitude of the in-plane magnetic induction (Figure 5D) peaks at 0.5 ± 0.1 T in the A2 gen-2 precipitates.

The Fe-Cr-Co-rich A2 gen-2 precipitates are surrounded by Fe-Co-rich A2 shells (Figure 4). The measured magnetic signal is therefore a superposition of contributions from the two A2 phases. Non-uniform magnetization distributions in some of the A2 precipitates may result from their “incomplete” morphologies in an ion-milled TEM specimen. 3D tomographic reconstruction was used to clarify the shapes and distributions of the A2 gen-2 precipitates in the B2 matrix from a tilt series of ADF STEM images (Midgley and Dunin-Borkowski, 2009). Figures 5E and S4 show sections through a tomographic reconstruction of region R1 that contains approximately spherical A2 gen-2 precipitates (yellow) in a B2 matrix (blue). Some of the precipitates intersect the TEM specimen surface and are incomplete.

#### *In situ* magnetic switching of BCC gen-2 precipitates in the B2 matrix (region R1)

**Figure 6:**
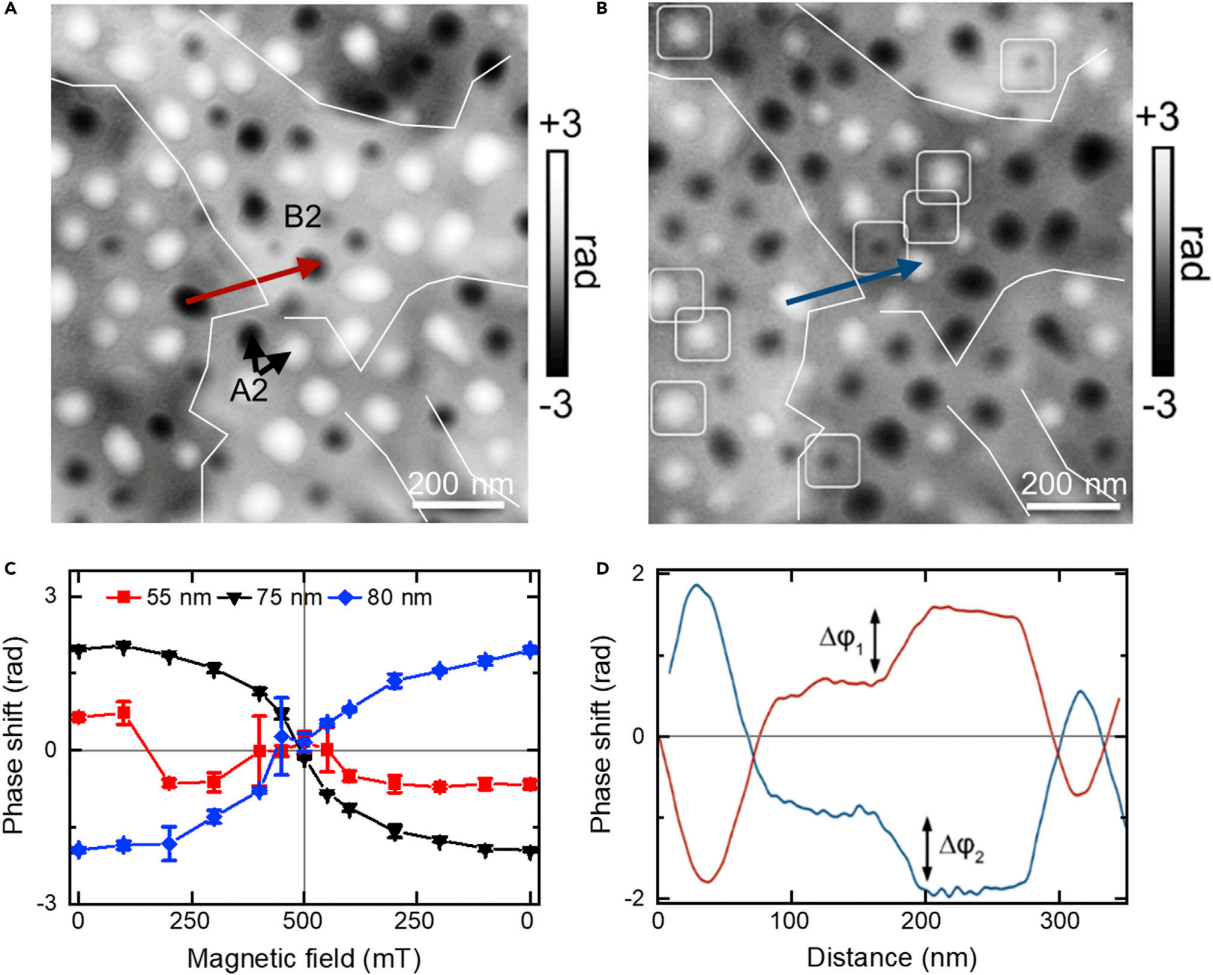
Magnetic switching of A2 gen-2 precipitates in the R1 phase in the AlCo_0.5_Cr_0.5_FeNi HEA (A and B) Magnetic contributions to the phase shift *φ*_*M*_ recorded after returning to remanence from opposite out-of-plane fields of −500 and 500 mT, respectively. The localized regions of dark and bright contrast correspond to clockwise or counterclockwise magnetization rotation directions in individual A2 precipitates. Contrast reversal of the A2 gen-2 precipitates is associated with a change in the magnetic field rotation direction of the vortices. In (B), marked precipitates (white squares) retained their magnetization rotation direction from that observed in (A). (C) Magnitude and sign of the local change in magnetic phase shift (*φ*_*M*_) of individual A2 gen-2 precipitates of different size plotted as a function of out-of-plane magnetic field. Positive values are associated with counterclockwise magnetization rotation directions. Error bars are the standard deviations of the phase shift. (D) Line profiles of magnetic phase shift extracted from (A and B) between two A2 gen-2 precipitates, as indicated by red and blue arrows in (A and B). The steps in phase shift Δ*φ* result from the presence of projected in-plane magnetic field contributions from the B2 matrix.

The magnetic switching properties of A2 gen-2 precipitates in the B2 matrix (region R1) were studied by applying magnetic fields perpendicular to the specimen plane. *In situ* magnetization reversal was performed by applying magnetic fields of up to 1.5 T using the conventional microscope objective lens. Figures 6A and 6B show magnetic phase images of region R1 recorded after returning to remanence from opposite out-of-plane fields of −500 and 500 mT, respectively. The proportion of clockwise and counterclockwise magnetic vortices in the A2 gen-2 precipitates in region R1 was measured from the magnetic phase images. A comparison of Figures 6A and 6B shows that the majority of the A2 gen-2 precipitates changed their magnetic field rotation direction. Within the field of view of approximately 1.4 *μ*m × 1.4 *μ*m, 46 counterclockwise and 34 clockwise vortices are visible in Figure 6A, whereas 35 counterclockwise and 45 clockwise vortices are visible in Figure 6B. White squares in Figure 6B mark precipitates that retained the same sense of magnetic rotation as in the initial remanent state.

Figure 6C shows the magnitude of the local change in magnetic phase shift *φ*_*M*_ recorded from representative A2 gen-2 precipitates of different size, plotted as a function of applied magnetic field from 0 → 500→ 0 mT. The positive (or negative) sign of *φ*_*M*_ is related to a counterclockwise (or clockwise) magnetic field rotation direction in the vortices. Two large A2 precipitates with diameters of 75 and 80 nm show a continuous decrease in magnetic phase shift to close to zero as the applied magnetic field is increased to 500 mT, suggesting that the internal field in the precipitates becomes aligned parallel to the electron beam direction. As the applied magnetic field is decreased (Figure 6C), the magnetic phase shift recovers, but with opposite sign, indicating that the magnetic vortex now has a rotation sense opposite to the original rotation direction. Different switching behavior was observed for a small A2 precipitate with a diameter of 55 nm (Figure 6C). The initial rotation sense is counterclockwise at 0 mT, changes sign at 200 mT, and decreases gradually to zero as the applied magnetic field increases to 500 mT. A possible scenario is that the magnetic field direction of the vortex core was aligned antiparallel to the saturating magnetic field. At 200 mT, the vortex core switches to become aligned with the saturating field, which also changes the vortex rotation direction. As the applied magnetic field is increased further, this state becomes aligned with the applied field direction and the magnetic phase shift approaches zero. On decreasing the applied magnetic field, a magnetic vortex forms again at 400 mT and remains stable as the applied magnetic field is reduced to zero.

The magnetic nature of the B2 matrix in region R1, which contains more than 60% Fe, Co, and Ni according to APT and EDXS measurements (Figure 4), is now discussed. Figure 6D shows a plot of the magnetic phase shift *φ*_*M*_ in the B2 matrix in region R1 before and after magnetization reversal, revealing a region with a gradient in phase and an associated step Δ*φ*_*M*_ of ∼1.3 rad. The greatest intensity maxima and minima in the plot correspond to A2 gen-2 magnetic vortices that changed their rotation direction during switching. These A2 phases serve as a reference for studying the magnetic phase contrast change within the matrix region. Changes in the sign of the magnetic phase shift *φ*_*M*_ indicate that part of the Al-Ni-Co-rich B2 matrix is also magnetized in the plane of the specimen, is ferromagnetic, and reverses in sign magnetically. Dipolar or magnetostatic interactions (Krishnan, 2016) between the A2 precipitates are expected to be affected by the nature of the surrounding magnetic phases and interphase boundaries. It is noteworthy that the same magnetic contrast is observed in the B2 matrix, but with a sign change in the magnetically switched region R1 (Figure 6).

The *in situ* magnetic switching experiments reveal details about the magnetic properties of region R1 in annealed AlCo_0.5_Cr_0.5_FeNi HEAs that contain A2-type precipitates in a B2 matrix. However, the lack of information about the core direction from off-axis EH experiments limits our understanding of the details of the process. The switching characteristics of the magnetic vortices depend on the sizes and shapes of the A2 gen-2 precipitates, the external magnetic field, and coupling to the B2 matrix.

#### Magnetic properties of AlCo_0.5_Cr_0.5_FeNi (II) – B2 precipitates in the A2 gen-1 matrix (region R2)

**Figure 7:**
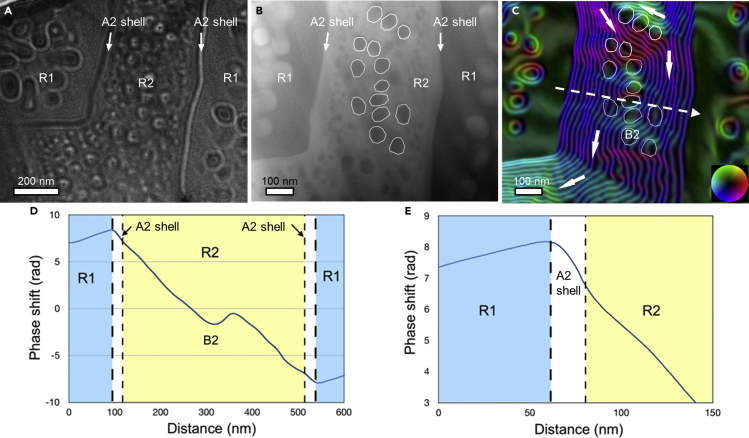
Magnetic domain structure of a B2 + A2 solid solution mixture in regions R1 and R2 and the A2 shell of the AlCo_0.5_Cr_0.5_FeNi HEA (A) Fresnel defocus image recorded at remanence at a defocus value of −200 *μ*m. (B) Mean inner potential contribution to the phase measured using off-axis EH from the R2 (+A2 shell) region between two R1 regions. (C) Corresponding magnetic induction map. The contour spacing is 2*π*/16 rad. Selected B2 inclusions in the A2 gen-1 matrix are marked with white frames in (B) and (C). The colors and arrows mark the projected in-plane magnetic field direction. (D) Line profile of the magnetic phase shift across an R2 region that includes A2 shell regions and a single B2 precipitate. The dip in the middle of the phase shift profile is associated with a weakly magnetic or non-magnetic region of B2. (E) Line profile of the magnetic phase shift, in which the slope in an A2 shell region (0.075 rad/nm) is 25% higher than that in an R2 region (0.06 rad/nm).

The AlCo_0.5_Cr_0.5_FeNi alloy contains regions (R2) of micrometer-sized Fe-Cr-Co-rich A2 gen-1 matrix with B2 precipitates. Microstructural and chemical studies show that an A2 shell is present between the B2 and A2 phases (Figure 4). Figure 7A shows a Fresnel defocus image of an R2 island surrounded by R1 recorded at a defocus value of −200 *μ*m. The magnetic nature of the R2 phase is apparent from the presence of dark and bright bands of contrast. The image also shows the limitation of Fresnel defocus imaging, as the phase boundaries give rise to strong fringing fields at their edges, making it difficult to interpret the magnetic state.

Figures 7B and 7C show the MIP contribution to the phase and a corresponding magnetic induction map recorded using off-axis EH. In Figure 7B, the A2 gen-1 and gen-2 (bright) and B2 (dark) phases can be distinguished, as they have different mean atomic numbers per unit volume. Selected B2 precipitates are marked by white frames. The color-coded magnetic induction map shows that the R2 region contains large magnetic domains. The magnetic field lines are disrupted or missing at the B2 precipitates, suggesting that they are weakly magnetic or non-magnetic. The effect of the smaller (<50 nm) B2 precipitates on the magnetic field is less clear, as it can be masked by the signal from the A2 gen-1 matrix in the ∼100-nm-thick TEM specimen.

Figure 7D shows a line profile of the magnetic phase shift across region R2, which contains a B2 precipitate at its center. The weakly magnetic or non-magnetic Al-Ni-Co-rich B2 precipitate has a lower contribution to the magnetic phase shift and appears as a dip. The phase profile in Figure 7E has a change in slope at the position of the A2 shell, suggesting a difference in its magnetic properties from the A2 gen-1 matrix. The phase gradients in the A2 shell and the A2 gen-1 matrix in region R2 are 0.075 and 0.06 rad/nm, respectively, based on fitted linear functions. As the magnetic phase shift scales with projected in-plane magnetic induction, it can be inferred that the A2 shell has ∼25% higher magnetization than the A2 gen-1 matrix in region R2. This difference is thought to result from the higher Cr concentration in the A2 gen-1 matrix, which decreases the magnetization of the Cr-Fe-Co-rich A2 gen-1 matrix in the R2 region.

## Discussion

Bulk measurements of structure and magnetic properties are not able to resolve and provide an understanding of the separate contributions of each phase. In contrast, the high spatial resolution analyses presented in this work reveal the intimate relationship between the structure and magnetic properties of the different phases. It is only then possible to rationalize the compositional effect on phase segregation, the elemental effect on the magnetic moment of each phase, and the contribution of constituent phases on the magnetic behavior.

### Compositional influence on phase segregation

The structural and magnetic properties of AlCo(Cr)FeNi HEAs are affected by the presence of non-magnetic Al and antiferromagnetic Cr. In AlCo(Cr)FeNi HEAs, Al has a favorable interaction with every other component, with particularly strong interactions with Ni and Co, which can be explained by the strengths of nearest-neighbor interaction energies, as determined using Monte Carlo simulations (Santodonato et al., 2018). This situation favors the formation of an AlNiCo phase. The nearest-neighbor-pair probabilities of elements in AlCo(Cr)FeNi HEAs show two characteristics: (i) strong ordering of Al, Co, and Ni with vanishing self-neighbor probability at low temperature; (ii) disordered segregation of Cr and Fe(Zhou et al., 2014). The Al-Ni-Co-rich phase possesses a B2 structure, since the B2 ordered structure has lower site ordering energy and spin ordering energy than a site-disordered BCC structure (A2) (Santodonato et al., 2018). These predictions are consistent with our experimental results and account for phase segregation in AlCo(Cr)FeNi HEAs.

### Compositional effect on magnetic properties in each phase

The saturation magnetic induction of AlCoFeNi and AlCo_0.5_Cr_0.5_FeNi is associated primarily with ferromagnetic ordering of Fe, Co, and Ni. According to current knowledge, Al atoms do not show magnetic moments in either the B2 or the A2 phase (Jung et al., 2019). In the Al-Ni-Co-rich B2 matrix, Ni atoms are very weakly magnetic in either the ferromagnetic (∼0.1 μ_B_ per atom) or the paramagnetic state (Santodonato et al., 2018; Zhou et al., 2014). The Co atoms are less magnetic in the ferromagnetic state (∼0.7 μ_B_ per atom) than in elemental Co, and their moments become even smaller in the completely spin-disordered (A2) paramagnetic state (Santodonato et al., 2018; Zhou et al., 2014). The Co atoms have a weak contribution to the magnetic moment of the phase. The Fe atoms have a much larger magnetic moment (∼2.9 μ_B_ per atom) in the B2 or L2_1_ ordered phase and have an almost similar moment to that in elemental Fe in the completely site-disordered phase (Santodonato et al., 2018; Zhou et al., 2014). In other words, in the Al-Ni-Co-rich B2 phase, site ordering increases the Fe moment and decreases the Co moment.

In the Fe-Cr-Co-rich A2 phase, Fe is the main magnetic element. Although the magnetic moments of Co atoms are approximately half of those of Fe atoms, their contribution to the total magnetic moment cannot be neglected (Cieslak et al., 2017). Jung et al. calculated the magnetic moments of all of the atoms in the B2, BCC (A2), and FCC phases in AlFeCoCrMn HEAs and came to similar conclusions (Jung et al., 2019). Fe and Co have high average magnetic moments, with higher average magnetic moments in the A2 phase than in the B2 phase.

In AlCoFeNi, our microstructural study reveals the presence of nanosized A2+B2 phases, while the replacement of Co by Cr results in greater B2+A2 phase segregation in the AlCo_0.5_Cr_0.5_FeNi alloy. Substitution of Co by Cr decreases the average magnetic moment as a result of its antiparallel magnetic ordering with Fe and Co in the A2 phase. Calculations of the partial density of states distribution show that Cr atoms in the BCC and FCC phases exhibit a more spin down than spin up distribution. Such distributions result in a reduction of the net magnetic moment owing to antiparallel alignments of the magnetic moments of Cr with respect to those of Fe and Co (Jung et al., 2019). In other words, the average magnetic moment of Cr atoms in the B2 phase is parallel to those of Fe and Co atoms, while the average magnetic moment of Cr atoms in the BCC and FCC phases is antiparallel to those of Fe and Co atoms. Schneeweiss et al. report the same conclusion: irrespective of the initial configuration of local magnetic moments, magnetic moments associated with Cr atoms align antiferromagnetically with respect to the cumulative magnetic moment of their first coordination shell and thus reduce the magnetic moment according to *ab initio* electronic density functional theory (Schneeweiss et al., 2017).

### The effect of constituent phases on magnetic properties of AlCo_0.5_Cr_0.5_FeNi HEAs

**Figure 8:**
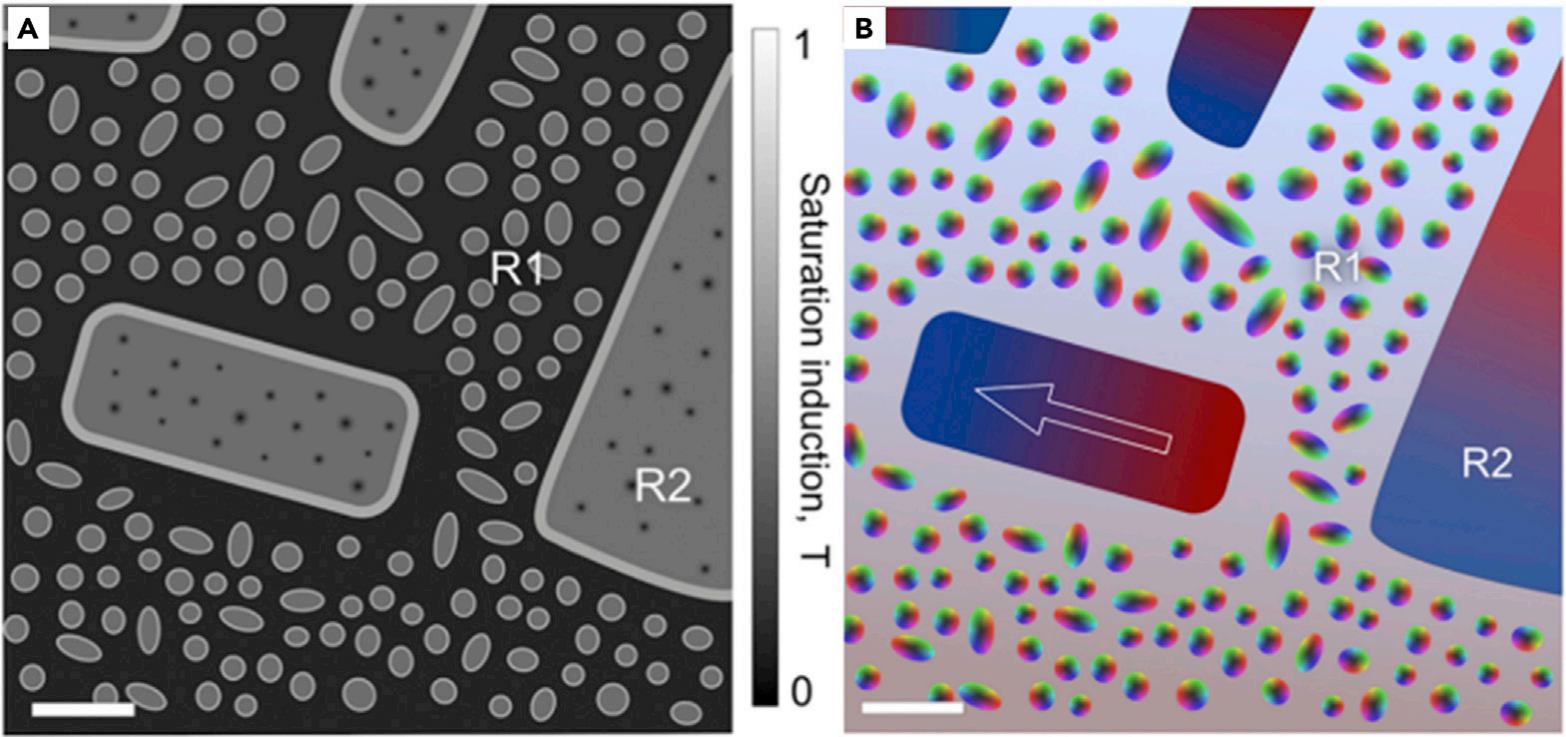
Schematic diagrams of magnetism of AlCo_0.5_Cr_0.5_FeNi (A and B) Schematic diagrams of (A) magnetic induction and (B) magnetic states in the phase-separated AlCo_0.5_Cr_0.5_FeNi HEA. The grayscale intensity in (A) corresponds to the deduced saturation magnetic induction in the different phases. The colors in (B) represent clockwise/counterclockwise magnetic field rotation in magnetic vortices, a slowly varying magnetic field in region 1 (R1), and large magnetic domains in region 2 (R2). The scale bar is 200 nm.

The constituent phases in AlCo_0.5_Cr_0.5_FeNi HEAs have different magnetic properties, resulting in rich and complex magnetic behavior. Based on the measurements in this work, schematic representations of the deduced saturation magnetic induction and magnetic states are presented in Figure 8. There are three primary contributions to the saturation magnetic induction:

(i) Fe-Cr-Co-rich A2 gen-2 and A2 gen-3 precipitates in region R1 and the A2 gen-1 matrix in region R2;
(ii) Fe- Co-rich A2 shells between the A2 and B2 phases;
(iii) the Al-Ni-Co-rich B2 phase.

The magnetic state of each phase is distinctly different. The first two contributions are strongly linked, as they form core-shell structures with thin A2 shells around A2 spheres or islands. Off-axis EH measurements reveal that the A2 shell in region R2 (Figure 7) has a higher magnetic induction than the A2 core (Figure 8A). Magnetic interactions, which can affect magnetization reversal, are expected between the two ferromagnetic A2 phases. The A2 spheres in region R1 support 3D magnetic vortex states in the B2 matrix. Based on the magnetic phase shift measurement and on the result of model-based iterative reconstruction of projected in-plane magnetization, the saturation magnetic induction in the A2 spheres, which have a core-shell structure, is estimated to be approximately 0.5 T (Figure 5). For a 2.6-nm-thick shell around a core with a radius of 40 nm, the shell occupies almost 20% of the total volume. Therefore, the contribution of the thin Fe-Co-rich A2 shell to the total magnetization is significant. A magnetic contribution is also expected from the B2 phase, which contains more than 50% Fe, Ni, and Co. In addition, the magnetic phase images (Figure 6) provide evidence for a slowly varying weak magnetic signal in the B2 matrix (Figure 8B). In a multicomponent and multi-phase alloy, one can estimate the effective value of Ms using an atomistic simulation based on density functional theory and by applying the rule of mixtures(Jung et al., 2019; Kao et al., 2011). However, it is likely that such a model cannot be used to describe coercivity.

In a multicomponent and multi-phase alloy, the coercivity *H*_*c*_ is expected to be sensitive to impurities, deformation, grain size, and phase decomposition (Herzer, 1990). For example, the coercivity of AlNiCo permanent magnets is known to depend on the nanostructure that develops during spinodal decomposition, involving an Fe-Co-rich phase and an Al-Ni-rich phase. The coercivity mechanism involves an interplay of size, chemistry, and possibly stress at interfaces (Zhou et al., 2014; Zhu et al., 2017). However, the relationship between these parameters and coercivity in HEAs that contain multiple phases on different length scales has rarely been studied.

The magnetic texture in the AlCoFeNi HEA (Figure 2) is characterized by large magnetic domains with small-angle magnetization variations. Magnetic domain walls move easily in the presence of an applied magnetic field. In contrast, in the AlCo_0.5_Cr_0.5_FeNi HEA, which has a multi-scale hierarchical B2+A2 decomposed structure, the A2 spheres in region R1 support 3D magnetic vortex states in the B2 matrix (Figure 5), with a random distribution of clockwise and anticlockwise field rotations. Based on these observations, magnetization reversal and domain wall movement across the different magnetic components are expected to be highly complex processes. Our magnetic switching study (Figure 6) shows that, in region R1, magnetic vortices in A2 gen-2 precipitates can change their rotation direction in the presence of external magnetic fields of 100–500 mT. The smaller the diameter of the A2 gen-2 precipitate, the lower is the magnetic field that is needed to change the magnetic vortex rotation direction, suggesting that the coercivity of such an AlCo_0.5_Cr_0.5_FeNi HEA can be tuned by changing the sizes and separations of the constituent A2 precipitates and islands. This prediction also matches well with the observation that nanosized A2+B2 phase separated AlCoFeNi shows a smaller value of *H*_*c*_ than that of the AlCo_0.5_Cr_0.5_FeNi alloy.

Our results provide direct information about the intricate complexity of magnetic remanent states and reversal processes in multicomponent HEAs that contain coexisting magnetic phases and hierarchical structures that span multiple length scales. The increased coercivity of the AlCo_0.5_Cr_0.5_FeNi HEA, which remains in the soft ferromagnetic range, can be attributed to a complicated magnetization reversal process, which involves the reversal of magnetic vortices in a weakly ferromagnetic matrix. Our results indicate that better soft magnetic properties (large Ms, small HC) can be obtained by optimizing the balance of the mass fraction of the A2 phase and the size of the A2 phase in AlCo(Cr)FeNi HEAs. It is therefore of interest for future studies of AlCo_0.5_Cr_0.5_FeNi magnetic HEAs to determine the effect of external magnetic field and/or stress annealing, kinetics, and other external stimuli on the formation of the precipitates and to achieve a control of magnetic anisotropy and magnetic properties, as already demonstrated successfully for AlNiCo. For instance, soft magnetic HEAs, in which moderate coercivity is tunable by magnetic nanostructures, are yet to be explored for high-frequency device applications.

### Limitations of the study

Our work described the correlation between the magnetic domain structure and constituent phases in a phase-separated high-entropy alloy. The magnetic imaging experiments have revealed the magnetic states at the remanence; however, the interactions between the different magnetic elements are remained unclear. *In situ* magnetizing experiments and micromagnetic simulations should address the dynamic and reversal processes. In addition, the effects of temperature, strain, and magnetic field on the formation of precipitates can be systematically investigated in the future to achieve control over the coercivity.

## STAR★Method

### Key resources table

**Table:** 

REAGENT or RESOURCE	SOURCE	IDENTIFIER
**Chemicals, peptides, and recombinant proteins**		

Co elemental powder	Alfa Aesar	CAS: 7413-34-5
Cr elemental powder	Alfa Aesar	CAS: 7440-47-3
Fe elemental powder	Alfa Aesar	CAS: 7439-89-6
Ni elemental powder	Alfa Aesar	CAS: 7440-02-0
Al elemental powder	Alfa Aesar	CAS: 7429-90-5
AlCoFeNi	This paper Borkar et al., 2017 Mendeley Data	https://doi.org/10.1002/adem.201700048
AlCo(Cr)FeNi	This paper Borkar et al., 2017 Mendeley Data	https://doi.org/10.1002/adem.201700048

**Deposited data**		

Related code for magnetization calculation	This paper	https://doi.org/10.5281/zenodo.6331522

**Software and algorithms**		

DigitalMicrograph	Gatan Inc	https://www.gatan.com/
HoloWorks	Gatan, Inc	https://www.gatan.com/products/tem-imaging-spectroscopy/holoworks-software
Semper	Saxton et al. (1979)	https://doi.org/10.1016/S0304-3991(79)80,044-3
Esprit	Bruker	https://www.bruker.com/en/products-and-solutions/elemental-analyzers/eds-wds-ebsd-SEM-Micro-XRF/software-esprit-family.html
IVAS 3.6.2	Cameca Instruments, Inc	https://www.cameca.com/service/software/ivas
Inspect 3D	Thermo fisher scientific	https://www.thermofisher.com/order/catalog/product/INSPECT3D?SID=srch-srp-INSPECT3D
Avizo	Thermo fisher scientific	http://www.thermofisher.com/amira-avizo
OriginLab	OriginLab Corporation	https://www.originlab.com
Python version 3.6	Python Software Foundation	https://www.python.org

**Other**		

TEM	Thermo fisher scientific	https://www.thermofisher.com/de/de/home.html
Fischione 2050	Thermo fisher scientific	https://www.thermofisher.com/de/de/home.html
Gatan K2 IS	Gatan, Inc	https://www.gatan.com/
FEI Helios Nanolab 400s	Thermo fisher scientific	https://www.thermofisher.com/de/de/home.html
FEI Nova Nanolab 20	Thermo fisher scientific	https://www.thermofisher.com/de/de/home.html
LEAP 3000X	Cameca Instruments, Inc	https://www.cameca.com/
VSM-Lakeshore 7404	Lake Shore Cryotronics, Inc	https://www.lakeshore.com/

### Resource availability

#### Lead contact

Further information and requests for resources and reagents should be directed to and will be fulfilled by the lead contact, Dr. Qianqian Lan (q.lan@fz-juelich.de).

#### Materials availability

This study did not generate new unique materials.

### Experimental model and subject details

Our study does not use experimental models typical in the life sciences.

### Method details

#### Specimen preparation

AlCo_*x*_Cr_1–*x*_FeNi (*x* = 0.5 and 1) bulk specimens were prepared by arc melting Al, Co, Cr, Fe and Ni pellets in an Ar atmosphere, followed by annealing at 600°C for 15 h in an Ar atmosphere and quenching in water (Borkar et al., 2017). Bulk magnetometry measurements were performed in a vibrating sample magnetometer (VSM-Lakeshore 7404) using a maximum magnetic field of 1 T. Specimens for TEM and APT were prepared using focused Ga ion beam milling in FEI Helios Nanolab 400s and FEI Nova Nanolab 20 dual beam systems using a standard lift-out method. Electron-transparent (∼100 nm) lamellae were attached to Cu Omniprobe support grids for TEM measurements.

#### Atom probe tomography

APT experiments were conducted in a LEAP 3000X local electrode atom probe system (Cameca Instruments, Inc.). All atom probe experiments were conducted in electric field evaporation mode at a temperature of 60 K using an evaporation rate of 0.5% and a pulsing voltage of 20% of the steady-state applied voltage. Data analysis was performed using IVAS 3.6.2 software.

#### Transmission electron microscopy

High-angle annular dark-field (HAADF) scanning TEM (STEM) imaging, energy-dispersive X-ray spectroscopy (EDXS) mapping and electron to-mography were performed in an FEI Titan G2 80-200 transmission electron microscope equipped with a high brightness field emission gun, a probe aberration corrector and an in-column Super-X EDXS system. HAADF STEM images were recorded on a Fischione detector using a beam convergence semi-angle of 24.7 mrad and an inner detector semi-angle of 69 mrad. The chemical compositions were analyzed using Esprit (Bruker) software.

#### 3D reconstruction of region R1 using STEM tomography

The three-dimensional shapes of the A2 gen-2 precipitates and their distribution in the B2 matrix in the AlCo_0.5_Cr_0.5_FeNi HEA were studied using ADF STEM tomography. A tilt series of ADF STEM images was recorded over a specimen tilt range of −70° to +70° using a tilt increment of 5° between -50° and +50° and 2.5° otherwise. The tilt series was recorded automatically using FEI tomography software version 4.0, with the dynamic focus function calibrated and enabled. Each ADF STEM image had a size of 2048 × 2048 pixels with a pixel size of 0.19 nm. The electron beam convergence semi-angle was 24 mrad and the inner collection semi-angle of the ADF detector was 43 mrad. The ADF STEM images were aligned using cross correlation and binned by a factor of 4. Tomographic reconstruction was carried out using the simultaneous iterative reconstruction technique with 100 iterations. Image alignment and reconstruction were performed using FEI Inspect3D software version 4.1.2. Visualization of the three-dimensional reconstruction was carried out using FEI Avizo software version 9.0.

#### Lorentz microscopy and off-axis electron holography

The same specimens that were used for microstructural characterization were used to study magnetic texture using Lorentz microscopy and off-axis EH. Off-axis electron holograms were recorded in magnetic-field-free conditions (*i.e.*, in Lorentz mode) in an image-aberration-corrected FEI Titan 80-300 transmission electron microscope equipped with a high brightness field emission gun, an electron biprism, and a direct electron counting detector (Chang et al., 2016) camera (Gatan K2 IS) using a typical exposure time of 6 s. The biprism voltage was typically set to 100 V, resulting in an overlap interference width of 2.1 *μ*m and a holographic interference fringe spacing of 2.76 nm with a contrast of 48% in vacuum. The objective lens of the microscope was used to apply out-of-plane magnetic fields to the specimen of between 0 and 1.5 T. Figure S5 shows an illustration of the approach used for off-axis electron holography experiments and data processing. The electrostatic and magnetic contributions to the phase shift were separated by turning the specimen over inside the electron microscope using a modified Fischione 2050 tomography specimen holder. Off-axis electron holograms were reconstructed numerically using a standard Fourier-transform-based method with sideband filtering using HoloWorks software in DigitalMicrograph in the Gatan microscopy suite, as well as using the Semper image processing language (Saxton et al., 1979). Contour lines and color maps were generated from recorded magnetic phase images to yield magnetic induction maps. OriginLab and Python were used to process the data.

### Quantification and statistical analysis

Values shown in Figure 6C are mean values obtained by multiple measuring the phase shift of individual A2 gen-2 precipitates of different size in each magnetic field using DigitalMicrograph. Error bars in Figure 6C are the standard deviations of the phase shift.

## Data Availability

All data reported in this paper will be shared by the lead contact upon request. All original code has been deposited at repository and is publicly available as of the date of publication. DOIs are listed in the key resources table. Any additional information required to reanalyze the data reported in this work paper is available from the Lead Contact upon request.
