# Supplementary material for: Highly complex magnetic behavior resulting from hierarchical phase separation in AlCo(Cr)FeNi high-entropy alloys
Source: iScience. 2022 Mar 11;25(4):104047. doi: 10.1016/j.isci.2022.104047 (PMC8961229; doi:10.1016/j.isci.2022.104047)
Supplement: Document S1. Figures S1–S5 [file mmc1.pdf]

iScience, Volume *25*

Supplemental information

Highly complex magnetic behavior resulting from hierarchical phase separation in AlCo(Cr)FeNi high-entropy alloys

Qianqian Lan, András Kovács, Jan Caron, Hongchu Du, Dongsheng Song, Sriswaroop Dasari, Bharat Gwalani, Varun Chaudhary, Raju V. Ramanujan, Rajarshi Banerjee, and Rafal E. Dunin-Borkowski

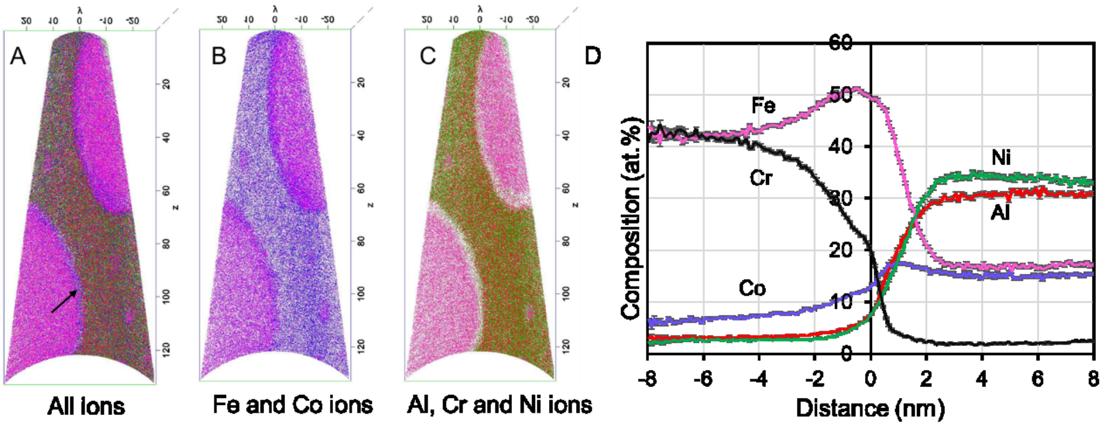

E

At.%	Al	Fe	Co	Ni	Cr	H
BCC gen-1	4.12±0.04	40.78±0.13	6.92±0.05	2.29±0.03	43.35±0.13	2.54±0.03
BCC gen-2	4.09±0.03	43.42±0.13	7.19±0.05	2.09±0.02	41.25±0.12	1.97±0.02
BCC gen-3	3.66±0.28	33.46±0.96	6.72±0.38	2.80±0.24	52.21±1.28	1.15±0.15
B2 phase	35.52±0.36	15.13±0.21	14.94±0.21	32.89±0.34	1.18±0.06	0.34±0.03

Figure S1. 3D atom probe tomography of region R1 in the $AlCo_{0.5}Cr_{0.5}FeNi$ HEA. (a-c) Reconstructions of A2 gen-2 and gen-3 precipitates in the B2 matrix in region R1. Fe-Co-rich shells form around the A2 gen-2 precipitates. Colors in the figure correspond to Al (red), Co (blue), Cr (black), Ni (green) and Fe (magenta). (d) Compositional profiles of Al, Co, Cr, Fe and Ni across A2 gen-2 precipitates and the B2 matrix. Fe and Co enrichment is visible at the interface region of the two phases. (e) Elemental compositions in the A2 gen-1, A2 gen-2 and A2 gen-3 regions and the B2 matrix measured using atom probe tomography. **Related to Figures 4A.**

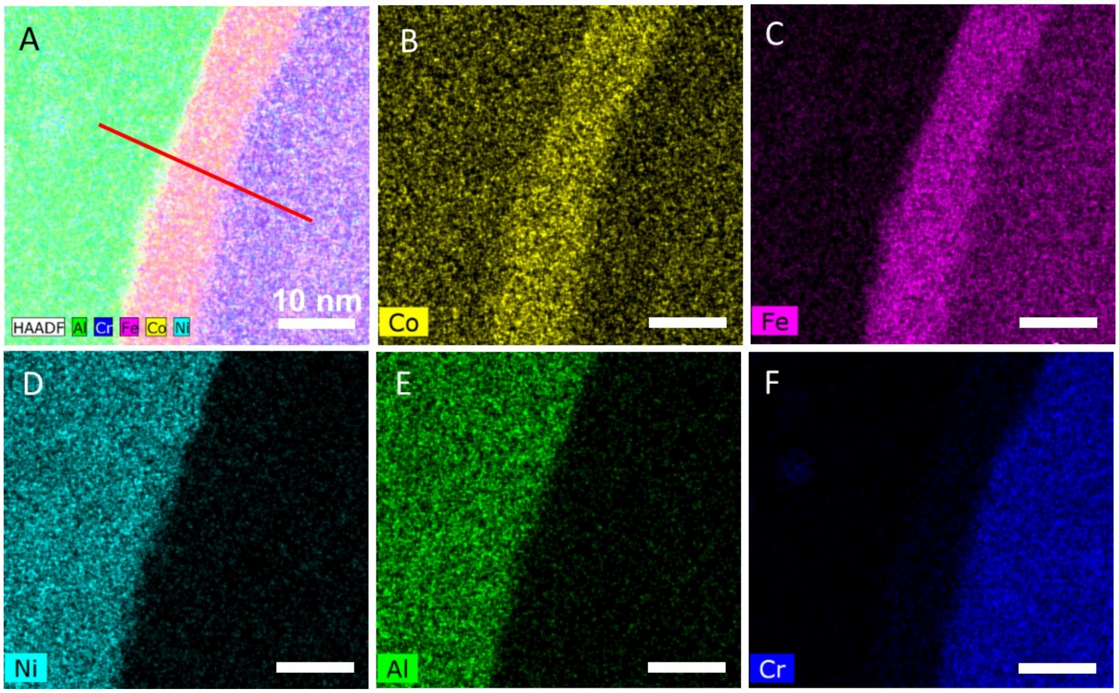

Figure S2. **Chemical composition of the A2 shell between the B2 phase in region R1 and the A2 gen-1 phase in region R2 in the $AlCo_{0.5}Cr_{0.5}FeNi$ HEA.** (a) Combined HAADF STEM image and Al, Cr, Fe, Co and Ni EDX maps. (b-f) Individual EDX maps for Co, Fe, Ni, Al and Cr. The scale bar in each image is 10 nm. **Related to Figure 4I.**

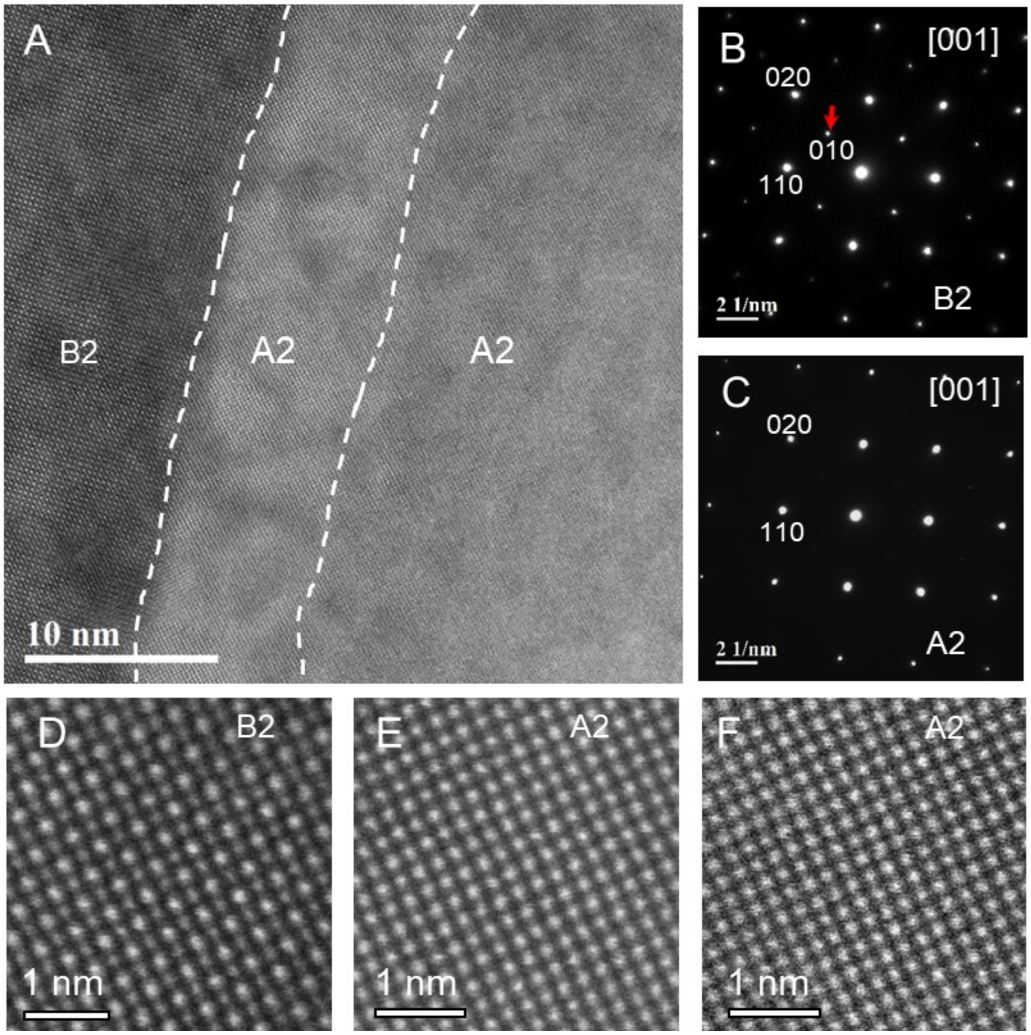

Figure S3. Microstructure of the A2 shell between the B2 phase in region R1 and the A2 gen-1 phase in region R2 in the $AlCo_{0.5}Cr_{0.5}FeNi$ HEA. (a) High-resolution HAADF STEM image of an R2 region and an A2 shell in an $AlCo_{0.5}Cr_{0.5}FeNi$ HEA, showing an approximately 7-nm-thick transition layer between A2 gen-1 islands and the B2 matrix. (b-c) Nano beam electron diffraction patterns recorded from regions containing the B2 phase and the A2 gen-1 phase. (d-f) Atomic-resolution HAADF STEM images of the B2 phase (lattice constant *a* is 0.288 nm) in region R1, the A2 shell (lattice constant *a* is 0.285 nm) and the A2 gen-1 phase (lattice constant *a* is 0.292 nm) in region R2, respectively. **Related to Figures 3 and Figure 4.**

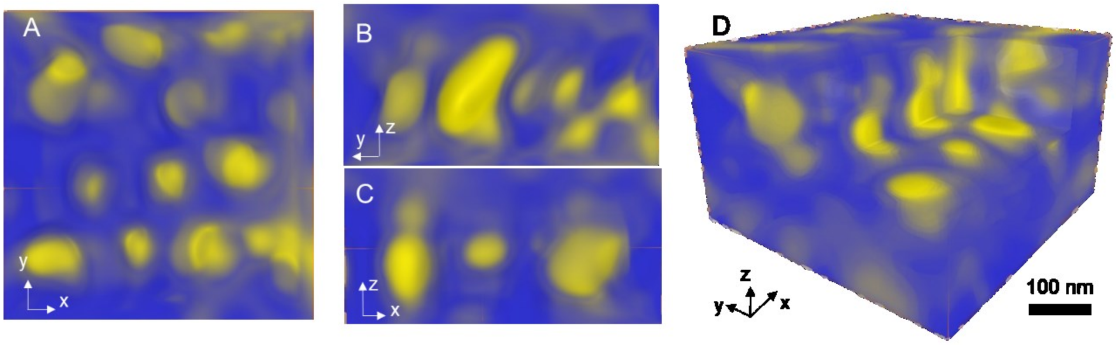

Figure S4. **Three-dimensional reconstruction of A2 gen-2 precipitates in the B2 matrix in region R1 using ADF STEM tomography.** The A2 gen-2 precipitates are shown in yellow, while the B2 matrix is shown in blue. The reconstruction is viewed from the top in (a), from the front in (b), from the left in (c) and in the form of a 3D visualization in (d). The A2 gen-2 precipitates (yellow) have close-to-spherical shapes, diameters of between 50 and 100 nm and are distributed randomly in the B2 matrix (blue). Figure S5d shows a 3D visualization of the reconstruction. **Related to Figures 5E.**

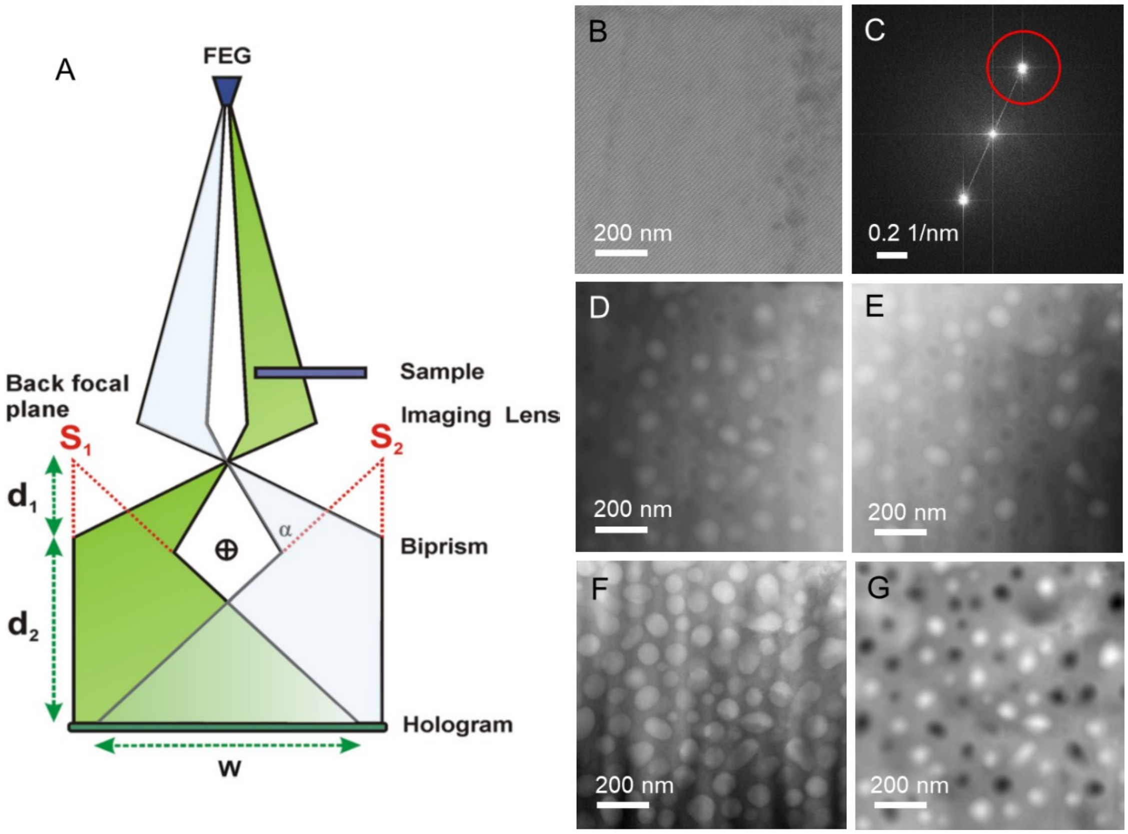

Figure S5. Illustration of approach used for off-axis electron holography experiments and data processing. (a) Schematic diagram of electron optical setup for off-axis electron holography. (b) Representative off-axis electron hologram recorded from region R1 in the $AlCo_{0.5}Cr_{0.5}FeNi$ HEA. (c) Fourier transform of the off-axis electron hologram shown in (b), containing a center band and two sidebands. A circular mask with smooth edges (marked by a red circle) was applied to one of the sidebands. (d) Reconstructed total phase shift image. (e) Similar reconstructed total phase shift image recorded from the same region after turning the sample over. (f) Mean inner potential contribution to the phase shift obtained by aligning the phase images in (d) and (e) in angle and position and evaluating half of their sum. (g) Magnetic contribution to the phase shift obtained by aligning the phase images in (d) and (e) in angle and position and evaluating half of their difference. **Related to Figures 5-7 and method.**
